# Spirit and Drug Taking among Doctors

**Published:** 1914-09

**Authors:** T. D. Crothers

**Affiliations:** Hartford, Conn.


### Spirit and Drug Taking Among Doctors.

BY T. D. CROTHERS, M. D., HARTFORD, CONN.

Hospitals and sanatoriums for the treatment of mental and nervous diseases report an increasing number of physicians suffering from drug and spirit addictions. Can it be possible that there is greater prevalence of these diseases among physicians than ever before?

Evidently the use of spirits as a beverage is growing less, and more physicians seem to be total abstainers at society meetings and at banquets. In much the same way the old-time drinking clergyman has disappeared, and the use of spirits on all festive occasions has passed away. One author writes very emphatically that the strains and stresses among medical men today are greater than ever, and that competition and poverty are accountable for the use of drugs and spirits among doctors, to relieve the conditions of stress.

Another reason which has not been mentioned is the faulty teachings of professors of therapeutics and the practice of medicine in many medical colleges, who still insist that alcohol is a safe stimulant in moderate quantities and a tonic to be relied upon; also, that opium and its alkaloids—cocaine and other narcotic drugs—are harmless and safe if only used with discretion.

In one of the prominent colleges a professor used to give most glowing lectures on the good effects of opium, cocaine and some of the coal-tar derivatives. He made little or no mention of the dangers, but extoled the remedial effects that would follow, and advised the students to experiment on themselves and thus be able to determine the value and limits of the drug. For a number of years, every class which graduated under this teaching, contributed a number of drug-takers, and finally the professor died from an overdose of morphia, having been a secret user for several years.

A teacher of the practice of medicine still working at his post, still urges alcohol as a safe tonic, and opium as most valuable. This man is making victims and actually training students to become inebriates and drug-takers.

Fortunately, these are rare examples. Teachers and professors in colleges have a great responsibility, if they do not make their students realize the danger from this source.

In all probability there are a great variety of causes of which faulty teaching is only one. Physicians who meet with success in the early part of their practice are very apt to become addicts under the mistaken notion that they need chemical help to enable them to do the work. Others who have great difficulty in building up a practice grow discouraged and for this find recourse in spirits and drugs. A third class cater to those who use spirits, and soon become like them, using spirits for real or imaginary causes.

The time is not far distant when a physician who is not a total abstainer, and who does not exemplify in his own life the highest type of both physical and mental health, will find great difficulty in securing a practice. Business men will demand the highest kind of mental efficiency possible in their medical advisers, and unless he measures up to this requirement the future will be difficult and dark.

